# A GAVeCeLT bundle for chest-port insertion: The SIC-Port protocol

**DOI:** 10.1177/11297298251375833

**Published:** 2025-11-12

**Authors:** Fabrizio Brescia, Maria Giuseppina Annetta, Fulvio Pinelli, Mauro Pittiruti

**Affiliations:** 1 Unit of Anesthesia and Intensive Care Medicine, Vascular Access Team, Centro di Riferimento Oncologico di Aviano, IRCCS, Aviano, Italy; 2 Department of Anesthesia and Intensive Care, Fondazione Policlinico Universitario “A. Gemelli” IRCCS, Rome, Italy; 3 Department of Anesthesia and Intensive Care, Careggi University Hospital, Firenze, Italy; 4 Department of Surgery, Fondazione Policlinico Universitario “A. Gemelli” IRCCS, Rome, Italy

**Keywords:** Ultrasound guidance, standardized assessment, central venous access, patient safety, centrally inserted central catheters, chest-port

## Abstract

The insertion of totally implantable venous access devices (ports) is a procedure widely used in clinical practice. Ports are generally used in cancer patients undergoing chemotherapy and in patients who require long-term, infrequent access (less than once a week). Over the past four decades, ports have been inserted primarily by direct cannulation of the deep veins of the supra/infra-clavicular area, with subcutaneous placement of the reservoir over the pectoralis major muscle (so-called “thoracic port” or “chest-port”). This paper describes an insertion bundle—developed by GAVeCeLT, the Italian Group of Long-Term Venous Access Devices, and nicknamed “SIC-Port” (Safe Insertion of Chest-Port)—which consists of few evidence-based strategies aiming to further minimize all immediate, early, or late complications potentially associated with chest-port insertion. The SIC-Port bundle is currently adopted by all training courses on chest-port insertion held by GAVeCeLT. It includes eight steps: (1) systematic ultrasound evaluation of the supra/infra-clavicular veins according to the Rapid Central Venous Assessment (RaCeVA) protocol, thus choosing the most appropriate vein, in terms of caliber, site, depth, safety of venipuncture, and ease of tunneling; (2) appropriate hand hygiene, proper skin antisepsis, and maximal barrier precautions; (3) ultrasound-guided cannulation of the best available vein, with preference for the axillary vein (if adequate), so to avoid tunneling above the clavicle; (4) ultrasound control of the pleura, to exclude pneumothorax; (5) ultrasound-guided tip navigation; (6) intra-procedural assessment of tip location by intracavitary ECG and/or trans-thoracic echocardiography with bubble test; (7) appropriate creation of the subcutaneous pocket over the muscle; (8) appropriate closure of the subcutaneous pocket.

## Introduction

The insertion of totally implantable venous access devices (or simply “ports”) is a widely used procedure in clinical practice. Ports are usually preferred in cancer patients undergoing chemotherapy and requiring long-term, infrequent access (less than once per week).^1–3^

In the last four decades, ports have been inserted mostly by direct cannulation of deep veins of the supra/infraclavicular area, with subcutaneous placement of the reservoir above the major pectoral muscle (so-called “chest-port”). Since its introduction in clinical practice in the 80s, chest-port insertion has been regarded as a relatively invasive procedure potentially associated with immediate complications (pneumothorax, hemothorax, arterial puncture, arrhythmias, air embolism) and late complications (thrombosis, occlusion, infection, catheter pinch-off).^4–6^

In the last two decades, many novelties have contributed to improve the safety of this clinical practice, the most important being the increasingly widespread use of ultrasound (US) in the different phases of chest-port insertion: preprocedural US evaluation of the deep veins of the arm, so to choose a vein of appropriate caliber; US-guided venipuncture using micro-introducer kits; immediate detection of possible puncture-related complications (such as tissue hematomas, intramural hematomas of the vein, pneumothorax, others). Ultrasound also allows for “tip navigation” (i.e. assessment of the correct direction of the guidewire and/or of the catheter while they progress into the vascular system), for “tip location” (i.e. assessment of the central position of the tip, by trans-thoracic echocardiography), and for the diagnosis of most late non-infective complications (fibroblastic sleeve, catheter-related venous thrombosis, tip migration, etc.).^3,7–14^

Ultrasound is of paramount importance, but is not the unique solution for the reduction of all catheter-related complications. Other evidence-based strategies are known to increase the safety and the cost-effectiveness of the procedure, reducing the risk of infection (hand hygiene, proper skin antisepsis, maximal barrier precautions), of insertion-related complications (adoption of micro-introduction kits), of venous thrombosis (use of 5–6 Fr catheters), of primary malposition (intraprocedural assessment of the location of the catheter tip, preferably by intracavitary ECG), and of mechanical complications of the pocket (preferential use of low-profile or very low profile reservoirs; appropriate choice of the site and dimension of the pocket, closure of the pocket by intradermal sutures and cyanoacrylate glue).^7–14^ At the same time, in the last two decades, the safety and cost-effectiveness of chest-port insertion has steadily increased thanks to the gradual abandonment of old strategies that were associated with potential damage to the patient and/or waste of resources: “blind” venipuncture based on surface landmarks, surgical isolation and cannulation of the vein, intraprocedural tip location by fluoroscopy, post-procedural tip location by chest X-ray, pocket closure by trans-cutaneous stitches.^14^

More recently, the use of brachial ports has also increased, thanks to the application of the technologies currently used for insertion of peripherally inserted central catheters (PICCs), bringing a new device into clinical practice, the PICC-port.^15,16^ Several studies have shown that insertion of PICC ports is safe (since it is not associated with any relevant immediate complication) and associated with optimal patient compliance, good cosmetic result, and low procedural costs; furthermore, the incidence of late complications (thrombosis, infection, occlusion, etc.) is similar for PICC-ports and chest ports.^15–18^ Nonetheless, there are specific contraindications to the insertion of PICC-ports (unavailability of arm veins of adequate caliber, previous dissection of axillary lymphatic nodes, stage 3b–5 chronic renal failure, paresis of the upper limb, vascular abnormalities, etc.), so that chest-ports still play a major role in the field of totally implanted venous access devices.

The GAVeCeLT (the Italian Group of Long-Term Venous Access Devices) has developed several protocols and bundles to standardize the insertion of venous access devices. Specific bundles have been proposed for the insertion of Centrally Inserted Central Catheters (CICCs),^19^ of Femorally Inserted Central Catheters (FICCs),^20^ of PICCs,^21,22^ and of PICC-ports.^23^

This paper aims to present a novel insertion bundle for chest-port, nicknamed “Safe Insertion of Chest-Port (SIC-Port).” The bundle includes all the technologies and methodologies that have contributed over the last 10 years to making chest-port insertion an increasingly safe and cost-effective procedure. It consists of eight different steps which correspond to evidence-based recommendations, taken from the most recent literature on the subject (Table 1).

**Table 1.** The eight steps of the SIC-port protocol.

Step 1	*Pre-procedural assessment*—systematic ultrasound examination of the veins of the cervico-thoracic region (according to the RaCeVA protocol) so to choose the most appropriate vein and plan the best location for the reservoir
Step 2	*Appropriate antiseptic technique*—strict policy of hand hygiene, skin antisepsis with 2% chlorhexidine in 70% isopropyl alcohol, and use of maximal barrier precautions
Step 3	*Ultrasound-guided venipuncture*—Ultrasound-guided cannulation of the best available vein, with preference for the axillary vein (if adequate), so to avoid tunneling above the clavicle
Step 4	*Ultrasound examination of the pleura*—rule out pneumothorax
Step 5	*Ultrasound-based tip navigation*—assess the correct direction of the guidewire and of the catheter by a supraclavicular ultrasound scan (according to the ECHOTIP protocol)
Step 6	*Intra-procedural assessment of tip location*—use intracavitary ECG and/or ultrasound-based tip location (subcostal or apical view, using the “bubble test”: according to the ECHOTIP protocol)
Step 7	*Appropriate subcutaneous placement of the reservoir*—subcutaneous placement of the reservoir above the major pectoral muscle, creating the pocket by hydro-dissection with local anesthetic and normal saline
Step 8	*Proper closure of the pocket*—closure of the skin incision with absorbable intradermal sutures and cyanoacrylate glue

RaCeVA: Rapid Central Vein Assessment; ECHOTIP: protocol of ultrasound-based tip navigation and tip location (see text).

### Preprocedural assessment

Proper pre-procedural assessment begins with an adequate anamnestic evaluation. It is important to assess whether the patients had a history of previous venous access devices, difficult venipuncture, or venous thrombosis. The coagulation status of the patient, as well as the use of antithrombotic therapies, should be considered before inserting the port.^24^ Specific contraindication to chest-port insertion should also be excluded. As for CICC insertion, bilateral contraindications include the evidence of an obstruction/compression of the superior vena cava (or of both innominate veins).

The most appropriate vein for cannulation should be chosen after a systematic ultrasound evaluation of the deep veins of the cervico-thoracic region.^19–23,25,26^ In this regard, it is wise to adopt the same protocol used before CICC insertion, the Rapid Central Vein Assessment protocol (RaCeVA), a systematic US evaluation of the veins of the neck and of the supra/infraclavicular area.^25^ The RaCeVA is designed as an easy, rapid, and systematic assessment of the six central veins that can be theoretically punctured and cannulated by US in the supra/infraclavicular area: internal jugular vein (IJV), external jugular vein (EJV), brachiocephalic vein (BCV), and subclavian vein (SV) in the supraclavicular area; axillary vein (AV) and cephalic vein (CV) in the infraclavicular area. US assessment of the deep veins of the cervico-thoracic area is performed using a 7–12 MHz linear transducer. With RaCeVA, the operator can rule out venous abnormalities such as thrombosis, stenosis, external compression, anatomical variations of size and shape of the veins, thus choosing a vein of appropriate caliber (ideally, at least three times the caliber of the catheter) so to reduce the risk of catheter-related thrombosis.^7,25,27,28^ Also, the RaCeVA allows for visualization of the surrounding arterial or nervous structures that could be accidentally injured during venous catheterization. The seventh and last step of RaCeVA is the US assessment of pleural space in the pre-insertion phase, providing an accurate baseline assessment of pleural function before venipuncture.^25^ In summary, with RaCeVA, the operator can consider all possible venous options, with the goal of accessing the most appropriate vein in terms of patient safety.

### Appropriate aseptic technique

For the whole procedure, after the first step of preprocedural assessment, the adoption of customized insertion packs is recommended, so to reduce manipulations, risks of bacterial contamination, and costs. A typical procedural pack for port will include the maximal barrier precautions, disposable surgical tools, syringes, needles, etc.^14^

The second step of the protocol addresses the antiseptic technique for minimizing the risk of bacterial contamination during the procedure.

Hand hygiene should be preferably performed by hydroalcoholic gel. In special cases, or when the hands are visibly dirty, the hydroalcoholic gel must be preceded by washing with antiseptic soap and water, according to current international guidelines on infection prevention.

For skin antisepsis prior to device insertion, 2% chlorhexidine in 70% isopropyl alcohol should be used.^29^ Iodine povidone, in either aqueous or alcohol solution, may have a role only in case of known allergy to chlorhexidine. Regarding the application technique of the antiseptic, no clinical difference in microorganism reduction between the concentric circle versus the back-and-forth techniques has been demonstrated when both techniques are used equally on clean and healthy skin.^30^

As recommended by all current guidelines, the risk of bacterial contamination must be reduced by adopting maximal barrier precautions: that is, non-sterile cap and facemask, sterile gown and gloves, full-size sterile drape over the patient, plus sterile cover for the ultrasound probe (long enough to cover both the probe and the cable when on the sterile field).^31^

These three cornerstones of infection prevention during insertion of venous access devices are the same recommended in the other insertion bundles developed by GAVeCeLT.^19–23^

### Ultrasound-guided venipuncture

The choice of the optimal vein to cannulate is crucial. As for CICC insertion,^19^ an essential parameter to consider is the inner diameter of the vein, which should be at least three times the outer diameter of the catheter. The intent is to maintain an ideal catheter-vein ratio (1:3 or less), so to reduce the risk of catheter-related thrombosis.^28,32^

Ultrasound-guided venipuncture is considered mandatory for any central venous catheterization.^3,7,10^ In the supraclavicular area, the IJV can be accessed by ultrasound guidance, preferably with vein visualization in short axis and in-plane puncture, so to minimize any risk of arterial injury. Other possible approaches in the supraclavicular are the ultrasound-guided venipuncture of BCV, SV, or EJV, with vein visualization in long axis and in-plane puncture.^25^ In the infraclavicular area, the axillary vein can be visualized in short axis, in long axis, or in oblique axis. The oblique axis view is obtained rotating the probe to almost halfway between the short axis and the long axis view: it allows the simultaneous visualization of axillary vein, axillary artery, pleura, ribs, and other surrounding structures, thus increasing the safety of the progression of the needle after in-plane puncture. The oblique axis + in-plane technique combines the advantages of the panoramic view with the optimal visualization of the needle tip obtained by the in-plane puncture.^33,34^ Regardless the technique of venipuncture, the use of a micro-introducer kits (21G echogenic needle, 0.018″ nitinol guidewire with straight soft tip, micro-introducer/dilator) is recommended because less invasive and less prone to bleeding complications if compared to 18–19 G needles and 0.035″ guidewires.^3,14,24^

**Figure 1.**
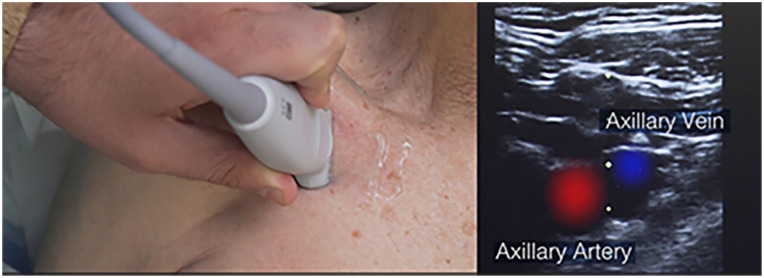
Ultrasound visualization of the axillary vein in the infraclavicular region.

Considering that for chest ports the reservoir is usually placed in the infraclavicular area, above the major pectoral muscle, the ultrasound-guided venipuncture of the axillary vein is ideal because it avoids the need of tunneling the catheter over the clavicle (Figure 1). If the axillary vein is not available for cannulation, tunneling is required so to move the catheter from the venipuncture site to the reservoir, which can be placed above the major pectoral muscle (in most cases) or above the biceps muscle (in selected cases).^14^ There are two main tunneling options: (a) tunneling from the supraclavicular area to the infraclavicular area (e.g. after venipuncture of the IJV or BCV), and (b) tunneling from the supra/infraclavicular area to the arm (e.g. after venipuncture of IJV, BCV, or AV).^14,19,35,36^ The latter type of tunnel might be useful, for example, in patients with indication to port insertion but with expected difficulty in placement of the reservoir in the infraclavicular area (presence of pacemaker or tunneled-cuffed catheter for dialysis, local alterations of the skin, scheduled radiotherapy of the chest area, voluminous breast implants, etc.); in these patients, if the insertion of a PICC-port is bilaterally contra-indicated by local or systemic issues (poor caliber of the veins of the upper extremity, long standing paresis with atrophy of the muscles, previous lymphatic dissection of the axilla, chronic renal failure requiring an arterial-venous fistula, etc.), the best choice is a “chest-to-arm” port, that is, a centrally inserted central catheter connected to a reservoir placed at the arm above the biceps muscle.^35,36^

Tunneling is preferably performed using blunt tunnelers, as they are associated with a minimal risk of local bleeding, even in patients with coagulation disorders or reduced platelet counts.^37^

### Ultrasound examination of the pleura

Soon after the US-guided venipuncture, US should also be used for ruling out pneumothorax, by detecting the “sliding sign” in the pleural space or other ultrasound signs that exclude the presence of pneumothorax, such as the “seashore sign” using M-mode.^27,38^ Both maneuvers can be performed with the same linear probe used for venipuncture.

Assessment of the absence of ultrasound signs suggestive of pneumothorax should be performed after any central venipuncture.^25^

### Ultrasound-based tip navigation

**Figure 2.**
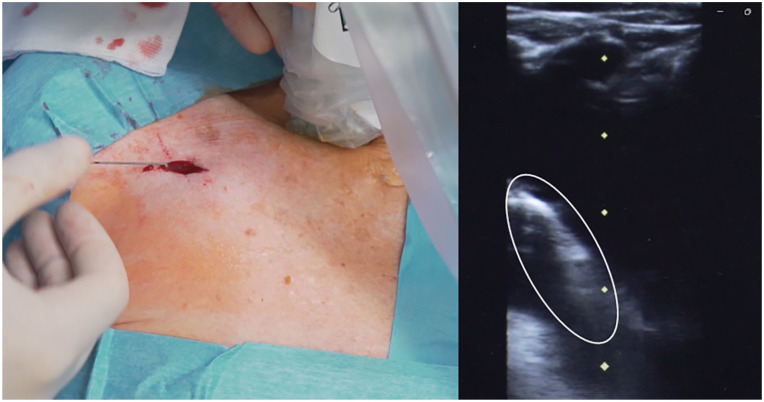
Ultrasound-based tip navigation: scan of the veins of the supraclavicular area according to the ECHOTIP protocol.

After catheter insertion into the introducer, ultrasound is also used for assessing the correct direction of the catheter toward the ipsilateral brachiocephalic vein (ultrasound-based “tip navigation”), by scanning the veins of the supraclavicular area. This maneuver can be performed with the same linear transducer used for the venipuncture, as described in the ECHOTIP protocol.^39,40^ Tip navigation with ultrasound has proven to be safer, easier, more widely applicable, and less expensive than fluoroscopy or electromagnetic tip navigation.^41^ Tip navigation may not always be necessary: if the position of the tip is rapidly verified, for example by intracavitary electrocardiography (see below), tip navigation may be redundant and time-consuming. On the contrary, if tip location is not immediately assessed, it is wise to verify if the catheter has taken the proper direction toward the brachiocephalic vein (Figure 2).

### Intra-procedural tip location

**Figure 3.**
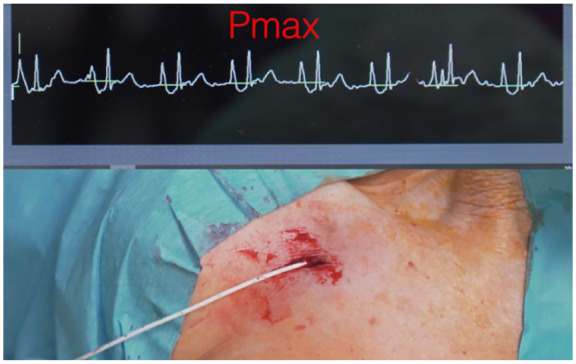
Intra-procedural tip location using intracavitary electrocardiography.

The central location of the catheter tip must be assessed during the insertion procedure: post-procedural verification and secondary adjustment of the catheter tip are overtly discouraged by current guidelines^3,7,10^ and are regarded as a waste of time and resources, not excluding potential harm to the patient. The safest, simplest, most cost-effective, and most accurate intra-procedural method for tip location is intracavitary electrocardiography (IC-ECG; Figure 3).^3,42^ Fluoroscopy-based tip location is inaccurate, expensive, logistically difficult, and inevitably unsafe because it implies exposure to ionizing radiation.^3,7,10,43^ The applicability of the IC-ECG method has also been recently extended to patients with atrial fibrillation.^44^ In patients with absent P wave not because of atrial fibrillation but because of other causes (pacemaker and/or implantable cardioverter-defibrillator; some rare arrhythmias; etc.), an effective, inexpensive, and non-invasive intraprocedural method for tip location is trans-thoracic echocardiography, preferably using the “bubble test,” according to a technique previously described in many studies.^7,45–47^ Ultrasound-based tip location requires a convex or sectorial transducer, using either a subcostal or apical view.^39,40,47^ However, this technique cannot replace IC-ECG in routine clinical practice, since it is somewhat less accurate, and it requires more training; also, its applicability/feasibility is sub-optimal in adult patients.^7,39,40,44^

### Appropriate creation of the subcutaneous pocket

An adequate subcutaneous pocket should be created, usually above the major pectoral muscle, preferably 2–3 cm from the head of the humerus, medial to the pectoral deltoid groove, no more than 2 cm below the clavicle. In some cases, the pocket will be created above the biceps muscle (chest-to-arm port); other potential locations for the reservoir are also to be considered, in very selected cases.^6,14^

The pocket should always be created with a “blunt” technique, by hydro-dissection (subcutaneous infusion of long-acting local anesthetic and/or normal saline), avoiding sharp tools, so as to minimize the risk of bleeding or injury to local structures. The height of the reservoir—usually low profile (approximately 10 mm high) or very low profile (approximately 8 mm high)—should be chosen based on the thickness of the subcutaneous tissue of the area where the pocket is created. Normal profile (12 mm) and high profile (14 mm) reservoirs are rarely indicated. For example, a very low-profile reservoir may be appropriate in a thin patient, or when the pocket is created on the arm (chest-to-arm). On the other hand, most patients with chest ports usually require a low-profile reservoir.^14^

After placing the reservoir into the subcutaneous pocket, the catheter is connected to the reservoir, and the proper function of the device (easy infusion of saline and easy aspiration of blood) is checked by accessing the reservoir with a Huber needle. It is advisable to trim the catheter 2 cm longer than the distance recorded by the IC-ECG, since 1 cm of catheter will be used for the connection to the reservoir, and the other extra cm will take into account that an approximate 1 cm sliding of the tip of the catheter far from the heart always occur when the patient moves from the supine to the upward posture.^14^

### Appropriate pocket closure

**Figure 4.**
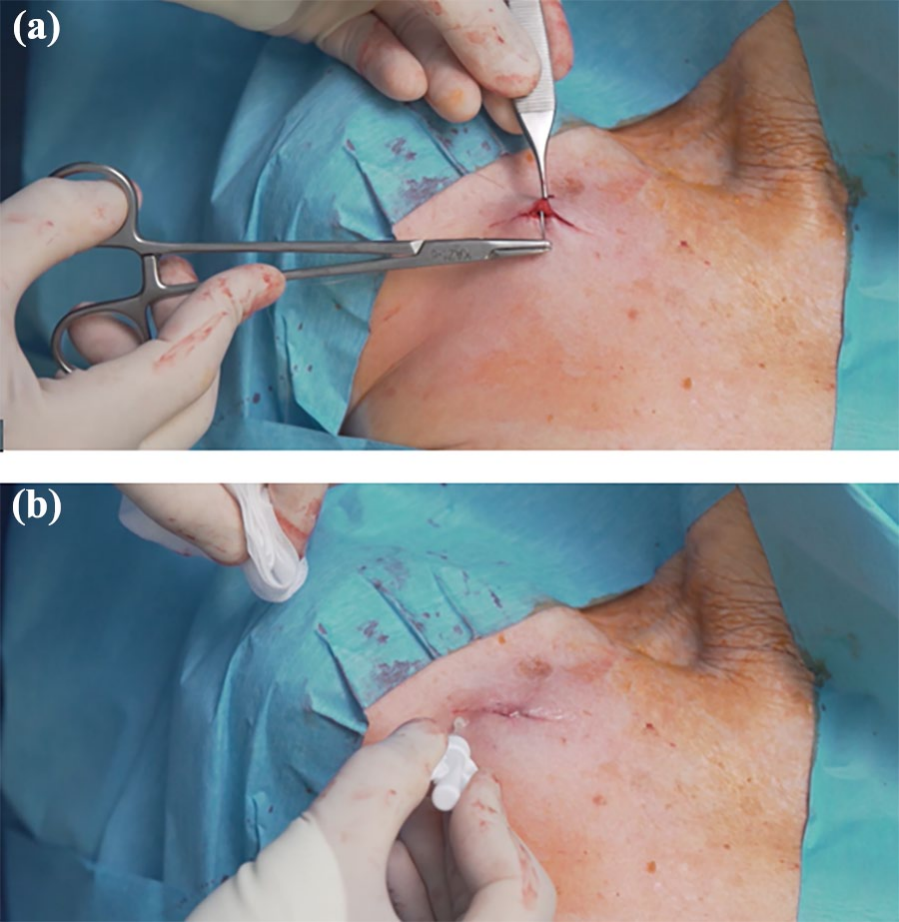
Appropriate pocket closure—the skin over the reservoir is closed with adsorbable, inverted intradermal sutures (a) and with cyanoacrylate glue (b).

The skin over the reservoir is closed with absorbable, inverted intradermal sutures and with cyanoacrylate glue (Figure 4). Such a technique of closure yields good cosmetic results of the scar and reduces the risk of infection.^48^ Skin closure and sealing with glue has been proven effective in several patient populations, from neonates to adults,^3,49,50^ in terms both of cosmetic result and of hemostatic and antimicrobial activity (Figure 4).^49–54^ A recent study on ports has investigated the closure of the skin comparing skin suture versus cyanoacrylate glue, finding that the latter technique did not increase the risk of local complications.^55^

After closure of the skin with cyanoacrylate, the patients must keep the wound covered for 3 days and avoid immersion in water for at least 1 week.

## Conclusions

An insertion protocol as the one above proposed, which consists in a bundle of evidence-based strategies, may facilitate the appropriateness of the maneuvers, and protect the patient from insertion-related complications, both immediate (puncture failure, arterial injury, hematoma, nerve damage, etc.) and late (infection, venous thrombosis, etc.). The correct implantation of the chest-port, respecting all these strategies, allows the immediate use of the device for any type of infusion.

The use of a standardized insertion bundle is always a clinician-friendly strategy: it saves time and resources, improves safety, and ensures cost-effectiveness. A consistent systematic adoption of all eight recommendations of the SIC-Port protocol may improve clinician performance while also providing a useful and evidence-based educational tool when teaching the fundamentals of chest-port insertion.
